# Emotion-Focused Human Resource Management and Compassionate Care Behavior: Integrating Emotion Regulation Theory With Public Service Motivation Theory

**DOI:** 10.1155/jonm/7902093

**Published:** 2025-10-15

**Authors:** Xiaodong Ji, Fei Wang, Ying Han, Dian Song, Yong Chen

**Affiliations:** ^1^Health Management Center, The First Affiliated Hospital of Soochow University, Suzhou, Jiangsu, China; ^2^Department of Political Science, School of Political Science and Public Administration, Soochow University, Suzhou, Jiangsu, China; ^3^Department of Administration, China Hulunbuir People's Hospital, Hulunbuir, Inner Mongolia, China; ^4^Department of Management Science, School of Political Science and Public Administration, Soochow University, Suzhou, Jiangsu, China; ^5^Department of Administration, Suzhou Science and Technology Town Hospital, Suzhou, Jiangsu, China

**Keywords:** compassionate care behavior, emotion-focused human resource management, emotional labor, public service motivation

## Abstract

**Aim:**

To examine the impact of emotion-focused human resource management (EFHRM) on compassionate care behavior (CCB) among nursing staff, and to explore the mediating role of emotional labor and the moderating effect of public service motivation.

**Background:**

CCB—nurses' empathetic and patient-centered actions that meet patients' emotional and physical needs—is essential for high-quality nursing care. However, its organizational antecedents remain underexplored.

**Methods:**

A cross-sectional survey was conducted among 272 nurses in hospital settings. EFHRM was assessed as a set of HR practices aimed at recognizing, supporting, and regulating employees' emotional needs and interpersonal sensitivities. Structural equation modeling was used to test the hypothesized relationships.

**Results:**

EFHRM has a significant and positive effect on nurses' CCB. Emotional labor partially mediated this relationship, indicating that HR practices addressing emotional needs enhance CCB by promoting emotionally engaged work. Moreover, public service motivation significantly moderated the mediation effect: nurses with higher levels of public service motivation were more likely to translate emotional labor into CCB. These results underscore both the psychological mechanisms and contextual factors that influence compassionate care delivery.

**Conclusions:**

The findings highlight the importance of EFHRM practices that are sensitive to the emotional dynamics of caregiving. EFHRM not only facilitates the emotional conditions necessary for CCB but also interacts with intrinsic motivational traits to enhance care quality. Understanding these processes offers valuable insights for healthcare organizations aiming to promote sustained, patient-centered care.

**Implications for Nursing Management:**

Organizations should adopt EFHRM strategies to create supportive emotional climates and cultivate compassionate care. Attention to both emotional labor and public service motivation can strengthen workforce resilience and patient outcomes in demanding care environments.

## 1. Introduction

Compassionate care behavior (CCB) refers to the actions taken by nurses to alleviate patients' physical and psychological discomfort, which involves understanding, making emotional responses, giving empathic care, and helping the patient [1, 2]. CCB significantly reduces the incidence of patient complications, accelerates patient recovery [3], and improves patients' satisfaction and well-being [4, 5]. Nurses are the primary, frontline implementers of CCB [6]. The enactment of CCB requires nurses to engage extensively in emotional labor, which can be classified into two distinct strategies: deep acting and surface acting [2, 7, 8]. Deep acting involves aligning expressed emotions with genuine internal feelings, whereas surface acting entails displaying emotions in accordance with organizational expectations rather than one's true emotional state [9]. Studies have shown that emotional labor has profound effects on various aspects of a nurse's professional life, including well-being [10], stress levels at work [11], job engagement [12], and their capacity for resilience [13]. Mitigating the adverse effects of emotional labor on CCB is a critical issue that deserves attention. However, the specific impact of emotional labor on the performance of CCB by nurses remains understudied [7].

To address emotional challenges, scholars have proposed the emotion-focused human resource management (EFHRM) model, which comprises recruitment and selection, training, performance appraisal, and reward management. EFHRM evaluates candidates' emotional regulation abilities, enhances employees' emotional competencies through training, and incorporates emotional performance metrics into evaluations. Additionally, it ties compensation to emotional performance, emphasizing the strategic role of emotional management in the workplace [14]. Nevertheless, research on EFHRM is still in its early stages. The overall impact of EFHRM on emotional labor and its specific influence on CCB remain insufficiently explored [14]. EFHRM primarily enhances nurses' emotional management and regulation skills, mitigating the negative effects of emotional labor and promoting CCB. This process can be explained through emotion regulation theory [15, 16]. According to emotion regulation theory, motivation plays a key role in determining the selection and implementation of emotion regulation strategies [17]. As providers of caring services, nurses should have a strong sense of public service motivation (PSM) [18, 19]. PSM can drive nurses to demonstrate a high degree of dedication and professional commitment in their nursing work [20]. Therefore, PSM provides a motivational basis for emotional labor management. The impact of emotional labor on CCB may be moderated by PSM [19].

**Figure 1:**
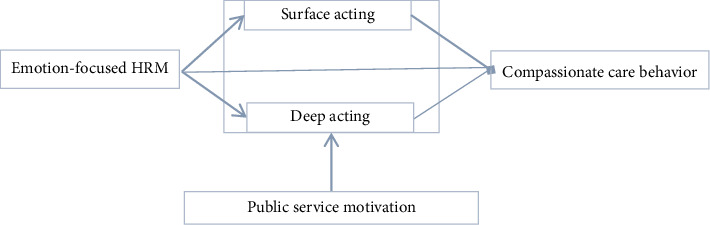
Research framework.

Therefore, drawing upon emotion regulation theory and PSM theory, we examine the relationships among EFHRM, emotional labor, CCB, and PSM. Specifically, the research aims to answer two questions. First, our study examines whether emotional labor serves as a mediator in the relationship between EFHRM and CCB behaviors. Second, the study explores the potential moderating influence of PSM on the mediating effect of emotional labor between EFHRM and CCB, assessing whether PSM intensifies or mitigates this relationship. Integrating the above two questions, we developed our research framework in Figure 1.

## 2. Hypothesis Development

### 2.1. EFHRM and CCB

Nurses, as frontline care providers, play a crucial role in delivering CCB, which not only alleviates patient suffering but also accelerates the recovery process [6]. These practices aid in easing patients' anxiety and contribute to cost reduction [21]. To effectively engage in CCB, nurses must develop the essential skill of empathetically understanding patients' emotional states. The EFHRM emphasizes the importance of selecting candidates with strong emotional recognition, regulation, and management abilities during the recruitment process [14]. The implementation of EFHRM requires hospitals to offer nurses comprehensive emotional management training, which includes both individualized courses and hands-on experience. Recruitment and training practices aim to cultivate a profound understanding of emotional awareness, enhance emotional acceptance skills, and improve the ability to manage impulsive reactions effectively [16]. Nurses' enhanced understanding of patients' emotions and needs, as supported by EFHRM's training, can enable them to provide more personalized and effective compassionate care [22]. This insight into patients' requirements is crucial in motivating nurses to undertake targeted CCB, which in turn helps alleviate patients' suffering [23].

During performance appraisals, hospitals focus on evaluating nurses' emotional display behaviors, including their emotional responses and emotion regulation strategies. Criteria for salary increases, and promotions are closely tied to nurses' emotional competencies [14]. Meanwhile, hospitals actively assist nurses in managing emotional dissonance and offer relaxation spaces to support emotional well-being. The institution's performance appraisal and reward systems are emotion-focused, effectively fostering strong motivation among nurses to engage in emotion regulation [22]. This proactive approach is crucial as it not only facilitates the expression of positive emotions toward patients but also leads to positive feedback, excellent performance evaluations, and increased rewards for the nurses [24]. In addition, EFHRM lays the foundation for nurses' capabilities and motivations to effectively implement CCBs. Empirical studies have demonstrated the profound influence of human resource management practices on CCB [22, 24, 25]. Therefore, we propose the following:

  H01: EFHRM does not positively affect CCB.
  H1: EFHRM positively affects CCB.

### 2.2. Mediating Role of Emotional Labor

Nurses' work is inherently intertwined with continuous patient interactions, making emotional labor a significant and integral aspect of their professional responsibilities [26]. As part of their emotional labor, nurses must regulate their emotional responses to meet the expectations and standards established by the healthcare organization [27]. In the course of their duties, nurses frequently face the challenge of managing their emotional expressions, especially when dealing with difficult patients or performing tasks they find unpleasant [28]. They must skillfully suppress their true feelings and adopt facial expressions that align with the organization's standards. This is known as surface acting, a form of emotional labor that is crucial for maintaining professionalism while protecting the well-being of both patients and nurses [29]. Conversely, when nurses engage with patients or tasks that evoke positive emotions, they can express genuine warmth and empathy through heartfelt smiles. This type of emotional expression represents deep acting, a form of emotional labor in which nurses internalize the required emotional state, ensuring that their displayed emotions align with their true feelings [30].

For surface-acting, nurses must employ antecedent-focused or response-focused strategies to effectively manage and suppress their emotions, ensuring their emotional expressions align with organizational expectations. In contrast, deep acting requires a more cognitive approach, where nurses reframe their perspective on their work, adjust their internal mindset, and genuinely convey concern for their patients through appropriate emotional responses [29]. Whether engaging in surface acting or deep acting, nurses require a robust capacity for emotional regulation. EFHRM plays a pivotal role in enhancing this capability and motivation. Initially, recruitment practices that emphasize emotional intelligence help hospitals identify and hire nurses with innate emotional management skills. Subsequently, targeted training and development initiatives further enhance these abilities. Finally, performance appraisal and reward systems that are attuned to emotional performance encourage adherence to the organization's emotional guidelines [14]. Therefore, we posit a positive correlation between EFHRM and the effectiveness of both surface and deep acting strategies.

Emotional labor significantly influences the attitudes and behaviors of employees within the workplace, shaping their professional activities and overall job satisfaction [31]. Surface acting results in negative outcomes such as nervousness, anxiety, and emotional exhaustion for employees, as it requires the continuous suppression of genuine emotions [20]. On the contrary, deep acting is associated with enhanced service quality, increased customer satisfaction, and improved employee performance, due to its alignment with employees' authentic feelings and a more engaged approach to their work [32, 33]. Nursing acts as an emotionally intricate profession, necessitating a significant degree of emotional labor to navigate complex interactions with patients and maintain a high standard of care [34]. Surface acting, which involves the suppression of one's natural emotional responses, can precipitate emotional exhaustion, psychological distress, and a range of adverse job-related experiences [35]. Consequently, when nurses engage in surface acting and suppress their natural emotional responses, they may struggle to empathize with patients' feelings and needs, reducing the likelihood of performing CCB. In contrast, deep acting fosters a profound connection with the work, enhancing job satisfaction and recognition [36]. This deeper emotional engagement significantly increases the propensity for nurses to engage in CCB, thereby improving the quality of care provided. Therefore, we argue that surface acting negatively relates to CCB, and deep acting positively relates to CCB. Combining with the relationship between EFHRM and CCB, the following hypothesis is proposed:

  H02a: Surface acting is not a mediator between EFHRM and CCB.
  H2a: Surface acting is a mediator between EFHRM and CCB.
  H02b: Deep acting is not a mediator between EFHRM and CCB.
  H2b: Deep acting is a mediator between EFHRM and CCB.

### 2.3. Moderating Role of PSM

Emotion regulation theory indicates that motivation is a key factor in an individual's emotional regulation [16]. The individual's beliefs, values, and goals influence the effectiveness of emotional regulation [37]. Emotional labor, as a strategy for emotion regulation, is shaped by an individual's motivations, including their inclination toward prosocial behavior and self-protective instincts [38]. For instance, reward motivation can effectively promote individual emotion regulation, and a high charitable trait has an implicit advantage in emotion regulation [39]. PSM is an individual's dynamic internal psychological tendency to benefit others and serve society [40, 41]. Emotional labor brings pressure to the nurse. However, individuals with high PSM regard pressure as their professional calling. With a profound sense of purpose in serving patients and society, these nurses actively immerse themselves in caregiving, often going above and beyond in their dedication. Thus, PSM acts as a crucial psychological resource, empowering nurses to navigate job stress and emotional challenges with greater resilience [42].

Therefore, when nurses possess a high level of PSM, the negative indirect effect of surface acting on CCB is weakened, as PSM offers psychological support to counteract the negative consequences of surface acting. On the other hand, the positive indirect effect of deep acting on such behaviors is strengthened by PSM, which imbues nurses with a sense of duty that fosters engagement in CCB. Accordingly, we propose the following hypothesis:

  H03a: The indirect relationship between surface acting and CCB is not moderated by PSM, the higher the PSM, the weaker the indirect relationship between surface acting and CCB.
  H3a: The indirect relationship between surface acting and CCB is moderated by PSM, the higher the PSM, the weaker the indirect relationship between surface acting and CCB.
  H03b: The indirect relationship between deep acting and CCB is not moderated by PSM, the higher the PSM, the weaker the indirect relationship between deep acting and CCB.
  H3b: The indirect relationship between deep acting and CCB is moderated by PSM, the higher the PSM, the weaker the indirect relationship between deep acting and CCB.

## 3. Methodology

### 3.1. Sample and Data Collection

The survey was conducted from May 2022 to December 2022 in Jiangsu Province, China. The respondents were nurses working in public hospitals. Participants were recruited through two primary channels. First, researchers based in hospitals contacted nursing staff directly via WeChat and invited them to complete the survey through the Questionnaire Star platform, a widely used and user-friendly survey tool in China. Second, using the snowball sampling method, hospital colleagues were asked to forward the survey invitation to nurses in other hospitals, who were also invited to complete the survey via the same platform. To ensure that all respondents were nursing staff, we adopted the following measures: First, when distributing the questionnaire, we explicitly requested that only nurses participate; second, the questionnaire included a clear statement indicating that only nursing personnel were eligible to complete it; third, even in cases where respondents held supervisory roles, we confirmed that they were also engaged in direct nursing work. Before beginning the survey, participants were presented with an introductory statement that served as both an information sheet and an informed consent form. This section clearly outlined the purpose of the study, the eligibility criteria (i.e., only nursing staff were invited to participate), and emphasized that the survey was anonymous and that no personally identifiable information would be collected. It also explicitly stated that participation was entirely voluntary. Participants were asked to read the consent statement carefully. Those who did not wish to participate could simply exit the questionnaire without completing it. The act of submitting a fully completed questionnaire was considered as providing informed consent to participate in the study.

To ensure consistency in participation across the three waves of data collection, each respondent was asked to provide the last four digits of their mobile phone number in every round, which were then used for matching. A total of 290 questionnaires were collected. Participants had the right to withdraw from the study at any time. Given the anonymous nature of the survey, no personally identifiable information was collected. Therefore, participants who wished to discontinue their involvement could do so by simply choosing not to complete the questionnaire. Incomplete responses were excluded from the final dataset. Responses with missing data at any of the three stages, or with phone numbers that could not be matched across waves, were excluded from the final sample. Eighteen such cases were identified and removed accordingly. After screening, 272 valid questionnaires were obtained. After screening, 272 valid questionnaires were obtained. This study was conducted in accordance with the Declaration of Helsinki. Retrospective ethical approval was granted by the Medical Ethics Committee of the First Affiliated Hospital of Soochow University (Approval No: (2024) Lun Yan Pi 527). Given the retrospective design and the anonymized nature of the dataset, the committee waived the requirement for informed consent.

The basic demographic information of the sample was presented in Table 1. The data collection was organized in three stages. During Stage 1, the nurse answered the questions about EFHRM and demographics. During Stage 2, about 2 months later, the nurse answered the questions about emotional labor and PSM. During Stage 3, about 2 months after Stage 2, the nurse answered the questions about CCB.

### 3.2. Measures

The items used a 5-point Likert scale ranging from “1 = *strongly disagree*” to “5 = *strongly agree*”. EFHRM includes five dimensions of recruitment, selection, training, performance evaluation, and reward systems with 15 items [14]. PSM adopts the scale developed by the authors in [41], which consists of 8 items. Emotional labor was measured by the scale developed by the authors in [43], which has 9 items. CCB was measured by the scale developed by the authors in [1], which consists of 7 items.

## 4. Analyses and Results

### 4.1. Validity and Reliability

Confirmatory factor analysis was conducted to assess the reliability and validity. It can be seen from Table 2 that the four-factor model is better than the one-factor, two-factor, and three-factor models. The results are as follows: X^2^/df = 2.84, CFI = 0.925, TIL = 0.911, RMSEA = 0.082, and SRMR = 0.052. It indicates that the criteria of the four-factor model met the requirements. Data analysis results indicate that the AVE value of each variable is greater than or equal to 0.665, the CR value is greater than or equal to 0.856, and Cronbach's alpha value of each variable is greater than or equal to 0.856. The loadings of each item, which are shown in Table 3, are higher than 0.50.

The means, standard deviations, and correlation coefficients of the variables in the study are demonstrated in Table 4.

### 4.2. Hypothesis Testing

#### 4.2.1. EFHRM and CCB

From Table 5, we can see that the regression coefficient of EFHRM on CCB is 0.598 (*p* < 0.01), which suggests that Hypothesis 1 is supported.

#### 4.2.2. Mediation Analysis

The PROCESS, developed by Hayes (2012), was used to test Hypotheses 2 and 3. We selected “Model 4” in PROCESS with a bias-corrected bootstrap analysis on 5000 resamples to calculate the indirect effects.  Table 6 indicates that the mediation effect of surface acting is 0.125 [0.070, 0.179], and the mediation effect of deep acting is 0.365[0.263, 0.467]. Beyond our expectations, EFHRM positively affects surface acting. Hypothesis 2 is partially supported.

#### 4.2.3. Moderated Mediation Analysis

To examine how the mediation effects of emotional labor in the relationship between EFHRM and CCB is contingent on PSM, we used “Model 14” in PROCESS with a bias-corrected bootstrap analysis on 5000 resamples to calculate the moderated mediation effects, where PSM was regrouped to three levels: mean—1 SD (low), mean ( medium), and mean + 1 SD (high). The results are shown in Table 7.

Although the indirect effects of surface acting between EFHRM on CCB are significant at each PSM level (low: *β* = 0.045, CI = [0.014, 0.109]; medium: *β* = 0.054, CI = [0.026, 0.094]; high: *β* = 0.064, CI = [0.023, 0.107]), the differences of emotion labor mediations among those PSM levels are significant. Similarly, the indirect effects of deep acting between EFHRM on CCB are significant at each EO level (low: *β* = 0.104, CI = [0.057, 0.163]; medium: *β* = 0.082, CI = [0.045, 0.131]; high: *β* = 0.060, CI = [0.029, 0.106]). The differences in deep-acting mediations among those PSM levels are significant. Therefore, H3 is partially supported.

Furthermore, regarding the moderating impact of PSM, we conducted two stages: the first stage and the second stage moderated mediation analysis. Concerning both surface and deep acting, we found that only first-stage moderated mediation is significant. The second stage and two stages of moderating mediation are not significant. Data analysis results are shown in Table 8.

**Figure 2:**
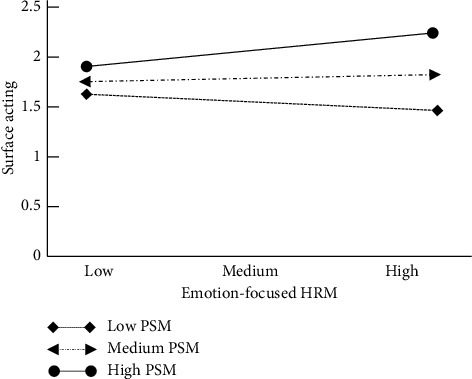
Moderation of PSM between EFHRM and surface acting.

**Figure 3:**
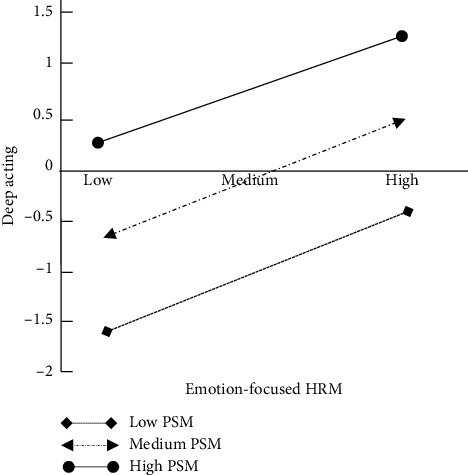
Moderation of PSM between EFHRM and deep acting.

Figures 2 and 3 show the first stage of moderating mediation effect.

## 5. Discussion and Conclusions

### 5.1. Major Findings

The major conclusions are as follows. First, EFHRM has a positive impact on healthcare workers' CCB. The conclusion is consistent with previous scholars' study [22]. Second, surface-acting and deep acting partially mediate the relationship between EFHRM and CCB. However, the negative effect of surface acting on CCB was not statistically significant. Third, PSM moderates the mediation effect of emotional labor in the relationship between EFHRM and CCB. When PSM is high, the mediation effect of deep acting decreases. The possible reason is that PSM has a significant direct impact on CCB. In contrast, when PSM is high, the mediation effect of the surface acting increases.

### 5.2. Theoretical Implications

This research contributes to the literature in three ways. First, few studies have analyzed the impact of EFHRM on employees' behavior [14]. Our study not only indicates the validity of EFHRM in the Chinese context but also demonstrates its positive effect on CCB, thereby enriching the antecedents of CCB. Second, as Grandey argued, emotion regulation is fundamental to the concept of emotional work. They conceptualize emotional labor as a process whereby individuals consciously regulate their feelings and expressions in order to align with organizational objectives [27]. Our research demonstrates that EFHRM can enhance the health workers' emotional labor management ability and boost their emotion regulation ability. Therefore, our study reveals the complex impact of emotional regulation ability on emotional labor, which deepens the theoretical research of emotional labor from the perspective of emotional regulation. Third, this study indicates that PSM moderates the indirect effect of emotional labor in the relationship between EFHRM practices and CCB. The conclusion further sheds light on the context in which emotional labor plays a role.

### 5.3. Managerial Implications

Practical implications are as follows. First, hospitals should widely implement the EFHRM system to enhance the health worker's service ability and direct them to provide better service quality. Second, hospitals should pay more attention to the emotional labor management of healthcare workers. Third, hospitals should improve healthcare workers' PSM. In daily work, organizations should establish fair and justice systems so that healthcare workers can feel that their contribution matches their reward, thus promoting their PSM of selfless dedication and inspiring more CCB from the heart.

### 5.4. Limitations and Further Research Directions

This study also has several limitations that need further improvement. First, one limitation of this study is the gender imbalance in the sample, as the vast majority of participants were female. However, this pattern is consistent with prior research findings, which have highlighted that the nursing workforce in China is predominantly female, with male nurses comprising only a small fraction. For example, scholar reported that in 2020, male nurses accounted for just 10.8% of the total nursing population in China [44]. Nonetheless, this limitation underscores the need for improved study design in future research, including greater efforts to recruit male nurses and explore potential gender-related differences in emotional labor and workplace behavior. Second, we only use questionnaire data, and most variable data are self-assessed by healthcare workers. Participants may answer the questions based on what they think is the most professional thing, rather than what they think, thereby leading to data collection bias. In the future, we can employ experimental data to test the hypothesis. Third, our research aimed to explore the impact of EFHRM practices on healthcare workers' CCB. However, we have only examined the mediating role of emotional labor. Based on the perspective of emotional competence, we can take variables such as emotional intelligence that reflect emotional competence as mediating variables to comprehensively examine the impact of EFHRM practices on healthcare workers' CCB in the future. Fourth, the hypothesized negative effect of surface acting on CCB was not supported by the empirical results. This outcome may be influenced by contextual factors such as cultural expectations, social desirability bias, or patients' limited ability to distinguish between emotional labor strategies. These potential explanations warrant further investigation in future research.

## Figures and Tables

**Table 1:** Sample.

Item	Types	Frequency	Percentages
Sex	Male	12	4.41
	Female	260	95.59

Age	Under 25 years of age	33	12.13
	26–35 years	130	47.79
	36–45 years	76	27.94
	46 years and above	33	12.13

Education level	Master's degree and above	6	2.21
	Undergraduate (adjective)	227	83.46
	Tertiary and below	39	14.34

Care worker level	Primary care workers	48	17.65
	Nurse practitioner	92	33.82
	The nurse practitioner in charge	98	36.03
	Deputy chief nursing officer	31	11.4
	Chief nursing officer	3	1.1

Number of years of nursing work	Less than 4 years	45	16.54
	5–9 years	76	27.94
	10–15 years	66	24.26
	16–20 years	40	14.71
	21 years and above	45	16.54

Total		272	100

**Table 2:** Measurement model.

Model	*X* ^2^	Df	*X* ^2^/df	RMSEA	IFI	TLI	CFI
1	1773.293	624.000	2.84182	0.082	0.925	0.911	0.925
2	3878.189	699.000	5.54819	0.129	0.793	0.779	0.792
3	6342.378	702.000	9.03473	0.172	0.632	0.610	0.631

*Note:* Model 1 is a combination of four variables; Model 2 combines public service motivation and emotional labor; Model 3 combines all variables.

**Table 3:** Items and loading.

Items	Loading
*Emotion-focused HRM*	
EFHRM1 applicant can accomplish emotional work	0.855
EFHRM2 applicant matches the company's value	0.921
EFHRM3 applicant maintains appropriate emotions during work	0.953
EFHRM4 applicant can accomplish emotion-related work	0.943
EFHRM5 applicant has a good fit with the company's culture	0.907
EFHRM6 training is consistent and integrated	0.797
EFHRM7 on-the-job training is aimed at how to respond properly	0.912
EFHRM8 training focus is on the ability to adapt to various conditions	0.917
EFHRM9 training to fit with the company's culture	0.942
EFHRM10 job rotation is often implemented	0.856
EFHRM11 focuses on proper responses	0.912
EFHRM12 multiple performance appraisals are used	0.907
EFHRM13 rewards based on the appropriate emotional states	0.964
EFHRM14 promotion based on appropriate emotions	0.968
EFHRM15 cares about emotions	0.924

*Emotional labor*	
EL1 I try to be kind to patients genuinely from my heart	0.891
EL2 I manage my expression and way of speaking with professional attitude to maintain patients' trust	0.958
EL3 I try to understand different circumstances between doctors and patients	
Patients	0.950
EL4 I try to overcome emotionally difficult situations with a sense of vocation as a nurse vocation as a nurse	0.968
EL5 I tolerate patients expressing negative emotions about medical staff or other departments to me or other departments to me	0.903
EL6 I tolerate unfair treatment to maintain a good work atmosphere on the ward	0.839
EL7 I pretend to feel what I don't actually feel when I deal with patients (e.g., empathy and interest) friendliness, delight)	0.725
EL8 I consciously control my facial expression, attitude, and way of speaking when interacting with other patients	0.890
EL9 although patients make me emotionally uncomfortable, I treat them with positive facial expressions and my attitude changes instantly	0.896

*Public service motivation*	
PSM1 meaningful public service activities are very important to me	0.914
PSM2 it is very important for me to contribute to the social welfare	0.978
PSM3 I think equal opportunity for all citizens is important	0.953
PSM4 the behavior of civil servants must conform to ethical rules	0.951
PSM5 it pains me to see other people in trouble	0.945
PSM6 it makes me angry when I see other people being treated unfairly	0.938
PSM7 the happiness of others matters	0.929
PSM8 I believe in putting civic duty before self-interest	0.878

*Compassionate care behavior*	
CB1 strive to understand your emotional needs	0.926
CB2 consider the effect of your illness on you, your family, and the people most important to you the people most important to you	0.970
CB3 listen attentively to you	0.966
CB4 convey information to you in an understandable way	0.932
CB5 always involve you in decisions about your treatment	0.897
CB6 comfortably discuss sensitive, emotional, or psychological issues	0.932
CB7 show respect for you, your family, and those important to you	0.799

**Table 4:** Correlation matrix.

	Variable	Mean	Std	1	2	3	4	5	6	7	8	9	10
1	Sex	1.96	0.21	1									
2	Age	2.40	0.85	0.12^∗^	1								
3	Edu	2.12	0.39	0.11^∗^	−0.01	1							
4	Grade	2.44	0.95	0.12^∗^	0.59^∗∗^	−0.28^∗∗^	1						
5	Year	2.87	1.32	0.13^∗^	0.87^∗∗^	−0.02	0.70^∗∗^	1					
6	EFHRM	3.90	0.84	0.08	−0.08	0.06	−0.09	−0.08	1				
7	SA	3.88	0.78	0.06	−0.03	−0.02	0.01	−0.02	0.67^∗∗^	1			
8	DA	4.31	0.68	0.04	0.02	0.001	0.03	0.06	0.68^∗∗^	0.66^∗∗^	1		
9	PSM	4.21	0.67	0.07	0.01	0.04	0.01	0.02	0.65^∗∗^	0.66^∗∗^	0.83^∗∗^	1	
10	CCB	4.23	0.68	0.03	0.00	0.04	0.03	0.03	0.72^∗∗^	0.74^∗∗^	0.89^∗∗^	0.88^∗∗^	1

Abbreviations: CCB, compassionate care behavior; DA, deep acting; EFHRM, emotion-focused human resource management; PSM, public service motivation; SA, surface acting.

^∗^ *p* < 0.1.

^∗∗^ *p* < 0.05.

^∗∗∗^ *p* < 0.01.

**Table 5:** Results of the main effect.

Dependent variable	CCB	
	Model 1	Model 2
Control variables		
Sex	0.061	−0.170
	(0.206)	(0.142)
Age	−0.082	−0.053
	(0.098)	(0.067)
Edu	0.075	0.039
	(0.116)	(0.080)
Grade	0.027	0.060
	(0.066)	(0.045)
Year	0.047	0.050
	(0.072)	(0.049)
Independent variable		
Emotion-focused HRM		0.598^∗∗∗^
		(0.034)
Cons	3.945^∗∗∗^	1.984^∗∗∗^
	(0.463)	(0.336)
*N*	272	272
*R* ^2^	0.006	0.536
Adj *R*^2^	−0.013	0.525
*F*	0.31	50.95

*Note:* Standard errors in parentheses.

^∗^ *p* < 0.1.

^∗∗^ *p* < 0.05.

^∗∗∗^ *p* < 0.01.

**Table 6:** Result of the mediation effect.

	Effect	Bootstrap SE	95% CI
Indirect surface acting	0.125	0.028	[0.070, 0.179]
Indirect deep acting	0.365	0.052	[0.263, 0.467]
Total indirect effect	0.490	0.056	[0.379, 0.600]
Direct effect	0.109	0.044	[0.023, 0.194]
Total effect	0.598	0.059	[0.484, 0.713]

*Note:* Total indirect effect is the sum of two mediating paths effects. CI is a confidence interval based on bias-corrected confidence intervals from bootstrap estimates; Bootstrap samples = 5000.

**Table 7:** Result of conditional indirect effect.

Mediators	Level of moderator	Conditional indirect effect	Bootstrap SE	95% CI
Surface acting	Low	0.045	0.024	[0.014, 0.109]
	Medium	0.054	0.017	[0.026, 0.094]
	High	0.064	0.022	[0.023, 0.107]

Deep acting	Low	0.104	0.027	[0.057, 0.163]
	Medium	0.082	0.022	[0.045, 0.131]
	High	0.060	0.020	[0.029, 0.106]

*Note:* Conditions for the moderator are the mean and/or minus one standard deviation from the mean. CI is a confidence interval based on bias-corrected confidence intervals from bootstrap estimates; Bootstrap samples = 5000.

**Table 8:** Result of moderation effect in the first stage.

Dependent variables	Model 3	Model 4	Model 5	Model 6	Model 7	Model 8
	Surface acting	Surface acting	Surface acting	Deep acting	Deep acting	Deep acting
Sex	0.268	0.019	−0.025	0.114	−0.110	−0.072
	(0.238)	(0.162)	(0.161)	(0.207)	(0.108)	(0.106)

Age	−0.048	−0.020	−0.013	−0.101	−0.077	−0.083^∗^
	(0.113)	(0.077)	(0.076)	(0.098)	(0.051)	(0.050)

Edu	−0.047	−0.102	−0.088	−0.013	−0.072	−0.084
	(0.134)	(0.091)	(0.090)	(0.117)	(0.061)	(0.060)

Grade	0.028	0.048	0.059	−0.016	−0.008	−0.018
	(0.076)	(0.052)	(0.052)	(0.067)	(0.035)	(0.034)

Year	−0.005	−0.009	−0.007	0.092	0.085^∗∗^	0.084^∗∗^
	(0.083)	(0.057)	(0.056)	(0.073)	(0.038)	(0.037)

Emotion-focused HRM		0.396^∗∗∗^	0.010		0.211^∗∗∗^	0.545^∗∗∗^
		(0.052)	(0.159)		(0.034)	(0.105)

PSM		0.461^∗∗∗^	0.179		0.683^∗∗∗^	0.927^∗∗∗^
		(0.064)	(0.127)		(0.043)	(0.084)

EFHRM^∗^ PSM			0.089^∗∗^			−0.077^∗∗∗^
			(0.035)			(0.023)

Con	3.520^∗∗∗^	0.538	1.738^∗∗∗^	4.131^∗∗∗^	0.942^∗∗∗^	−0.096
	(0.534)	(0.404)	(0.614)	(0.466)	(0.269)	(0.406)

*R* ^2^	0.007	0.547	0.558	0.008	0.736	0.747

Adj *R*^2^	−0.011	0.535	0.544	−0.010	0.729	0.739

*F*	0.39	45.50	41.49	0.45	105.19	97.07

*Note:* Standard errors in parentheses.

^∗^ *p* < 0.1.

^∗∗^ *p* < 0.05.

^∗∗∗^ *p* < 0.01.

## Data Availability

The data supporting the findings of this study are available from the corresponding author upon reasonable request.

## Supporting Information

This attachment is an ethical approval certificate issued by the Medical Ethics Committee of the First Affiliated Hospital of Soochow University, which certifies that the study has been conducted in compliance with relevant laws and regulations of China as well as international ethical guidelines. The ethical approval number is (2024) Ethics Research No. 527.

## References

[1] Lown B. A., Muncer S. J., Chadwick R. (2015). Can Compassionate Healthcare Be Measured? the Schwartz Center Compassionate Care Scale. *Patient Education and Counseling*.

[2] Su J. J., Masika G. M., Paguio J. T., Redding S. R. (2020). Defining Compassionate Nursing Care. *Nursing Ethics*.

[3] Hojat M., Louis D. Z., Markham F. W., Wender R., Rabinowitz C., Gonnella J. S. (2011). Physicians’ Empathy and Clinical Outcomes for Diabetic Patients. *Academic Medicine*.

[4] Lown B. A., Dunne H., Muncer S. J., Chadwick R. (2017). How Important is Compassionate Healthcare to You? A Comparison of the Perceptions of People in the United States and Ireland. *Journal of Research in Nursing*.

[5] Post S. G. (2011). Compassionate Care Enhancement: Benefits and Outcomes. *The International Journal of Person Centered Medicine*.

[6] Sinclair S., Hack T. F., Raffin-Bouchal S. (2018). What Are Healthcare Providers’ Understandings and Experiences of Compassion? the Healthcare Compassion Model: A Grounded Theory Study of Healthcare Providers in Canada. *BMJ Open*.

[7] Msiska G., Smith P., Fawcett T., Nyasulu B. M. (2014). Emotional Labour and Compassionate Care: What’s the Relationship?. *Nurse Education Today*.

[8] Rankin B. (2013). Emotional Intelligence: Enhancing Values-Based Practice and Compassionate Care in Nursing. *Journal of Advanced Nursing*.

[9] Grandey A. A. (2003). When ’The Show Must Go On’: Surface Acting and Deep Acting as Determinants of Emotional Exhaustion and Peer-Rated Service Delivery. *Academy of Management Journal*.

[10] Chou H. Y., Hecker R., Martin A. (2012). Predicting Nurses’ Well-Being From Job Demands and Resources: A Cross-Sectional Study of Emotional Labour. *Journal of Nursing Management*.

[11] Karimi L., Leggat S. G., Donohue L., Farrell G., Couper G. E. (2014). Emotional Rescue: The Role of Emotional Intelligence and Emotional Labour on Well-Being and job-Stress Among Community Nurses. *Journal of Advanced Nursing*.

[12] Mauno S., Ruokolainen M., Kinnunen U., De Bloom J. (2016). Emotional Labour and Work Engagement Among Nurses: Examining Perceived Compassion, Leadership and Work Ethic as Stress Buffers. *Journal of Advanced Nursing*.

[13] Delgado C., Evans A., Roche M., Foster K. (2022). Mental Health Nurses’ Resilience in the Context of Emotional Labour: An Interpretive Qualitative Study. *International Journal of Mental Health Nursing*.

[14] Chen L.-H., Lin S.-P. (2009). Reducing Service Agents’ Emotional Labor by Emotion-Focused Human Resource Management Practices. *Social Behavior and Personality: An International Journal*.

[15] Gross J. J. (1998). Antecedent- and Response-Focused Emotion Regulation: Divergent Consequences for Experience, Expression, and Physiology. *Journal of Personality and Social Psychology*.

[16] Gratz K. L., Roemer L. (2004). Multidimensional Assessment of Emotion Regulation and Dysregulation: Development, Factor Structure, and Initial Validation of the Difficulties in Emotion Regulation Scale. *Journal of Psychopathology and Behavioral Assessment*.

[17] Tamir M., Schwartz S., Oishi S., Kim M. (2017). The Secret to Happiness: Feeling Good or Feeling Right?. *Journal of Experimental Psychology: General*.

[18] Perry J. L., Wise L. R. (1990). The Motivational Bases of Public Service. *Public Administration Review*.

[19] Potipiroon W., Srisuthisa-Ard A., Faerman S. (2019). Public Service Motivation and Customer Service Behaviour: Testing the Mediating Role of Emotional Labour and the Moderating Role of Gender. *Public Management Review*.

[20] Roh C.-Y., Moon M. J., Yang S.-B., Jung K. (2016). Linking Emotional Labor, Public Service Motivation, and Job Satisfaction: Social Workers in Health Care Settings. *Social Work in Public Health*.

[21] Zeray M., Mariam D. H., Sahile Z., Hailu A. (2021). Validity and Reliability of the Amharic Version of the Schwartz Center Compassionate Care Scale. *Plos One*.

[22] Parveen K., Hussain K. A.-O., Afzal M., Gilani S. A. (2020). Determining the Association of High-Commitment Human Resource Practices With Nurses’ Compassionate Care Behaviour: A Cross-Sectional Investigation. *Journal of Nursing Management*.

[23] Izard C., Stark K., Trentacosta C., Schultz D. (2008). Beyond Emotion Regulation: Emotion Utilization and Adaptive Functioning. *Child Development Perspectives*.

[24] Zamanzadeh V., Valizadeh L., Rahmani A., van der Cingel M., Ghafourifard M. (2018). Factors Facilitating Nurses to Deliver Compassionate Care: A Qualitative Study. *Scandinavian Journal of Caring Sciences*.

[25] Koole S. L., Fockenberg D. A. (2011). Implicit Emotion Regulation Under Demanding Conditions: The Moderating Role of Action Versus State Orientation. *Cognition & Emotion*.

[26] Isbell L. M., Tager J., Beals K., Liu G. (2020). Emotionally Evocative Patients in the Emergency Department: A Mixed Methods Investigation of Providers’ Reported Emotions and Implications for Patient Safety. *BMJ Quality and Safety*.

[27] Grandey A. A. (2000). Emotional Regulation in the Workplace: A New Way to Conceptualize Emotional Labor. *Journal of Occupational Health Psychology*.

[28] Shaqiqi W., Smith P., Shaqiqi R. (2024). Exploring the Emotional Labour of Paediatric Oncology Nurses and Its Impact on Their Well-Being: An Integrative Review. *European Journal of Oncology Nursing*.

[29] Hwang W. J., Park E. H. (2022). Developing a Structural Equation Model From Grandey’s Emotional Regulation Model to Measure Nurses’ Emotional Labor, Job Satisfaction, and Job Performance. *Applied Nursing Research*.

[30] Saei E., Lee R. T. (2024). Psychological Hardiness, Social Support, and Emotional Labor Among Nurses in Iran During the CoviD-19 Pandemic: A Cross-Sectional Survey Study. *International Journal of Nursing Studies Advances*.

[31] Wong J. Y., Wang C. H. (2009). Emotional Labor of the Tour Leaders: An Exploratory Study. *Tourism Management*.

[32] Kammeyer‐Mueller J. D., Rubenstein A. L., Long D. M. (2013). A Meta‐Analytic Structural Model of Dispositonal Affectivity and Emotional Labor. *Personnel Psychology*.

[33] Wang H., Hall N. C., Taxer J. L. (2019). Antecedents and Consequences of Teachers’ Emotional Labor: A Systematic Review and Meta-Analytic Investigation. *Educational Psychology Review*.

[34] Pisaniello S. L., Winefield H. R., Delfabbro P. H. (2012). The Influence of Emotional Labour and Emotional Work on the Occupational Health and Well-Being of South Australian Hospital Nurses. *Journal of Vocational Behavior*.

[35] Humphrey R. H., Pollack J. M., Hawver T. (2008). Leading With Emotional Labor. *Journal of Managerial Psychology*.

[36] Zaghini F., Biagioli V., Proietti M., Badolamenti S., Fiorini J., Sili A. (2020). The Role of Occupational Stress in the Association Between Emotional Labor and Burnout in Nurses: A Cross-Sectional Study. *Applied Nursing Research*.

[37] Tamir M., Ford B. Q. (2012). When Feeling Bad is Expected to be Good: Emotion Regulation and Outcome Expectancies in Social Conflicts. *Emotion*.

[38] Grandey A. A., Melloy R. C. (2017). The State of the Heart: Emotional Labor as Emotion Regulation Reviewed and Revised. *Journal of Occupational Health Psychology*.

[39] Benita M. (2020). Freedom to Feel: A Self-Determination Theory Account of Emotion Regulation. *Social and Personality Psychology Compass*.

[40] Perry J. L. (2014). The Motivational Bases of Public Service: Foundations for a Third Wave of Research. *Asia Pacific Journal of Public Administration*.

[41] Bao Y., Li C. (2016). Measuring Public Service Motivation: Theoretical Structure and Scale Revision. *Human Resources Development of China*.

[42] Bakker A. B. (2015). A Job Demands-Resources Approach to Public Service Motivation. *Public Administration Review*.

[43] Yao Y., Wei W., Hu Y., Zhang Y., Chen M. (2020). Translation and Psychometric Validation of the Chinese Version of the Emotional Labour Scale for Nurses. *Journal of Nursing Management*.

[44] Xu H., Liang C., Kong J., Chen Q., Zhao Y., Zhang F. (2024). Reliability and Validity Evaluation of the Chinese Version of the Gender Misconceptions of Men in Nursing (Gemini) Scale Among Nursing Students. *BMC Nursing*.

