# Supplementary material for: Emotion-Focused Human Resource Management and Compassionate Care Behavior: Integrating Emotion Regulation Theory With Public Service Motivation Theory
Source: J Nurs Manag. 2025 Oct 15;2025:7902093. doi: 10.1155/jonm/7902093 (PMC12543497; doi:10.1155/jonm/7902093)
Supplement: Supporting Information — Additional supporting information can be found online in the Supporting Information section. [file 7902093.f1.pdf]

苏州大学附属第一医院医学伦理委员会
回顾性/非干预性研究快速审核表

审核编号：（2024）伦研批第 527 号

研究项目名称	情绪聚焦型人力资源管理与同理关爱行为：情绪调节与公共服务动机的整合研究
研究类别	回顾性研究
项目来源	自筹
承担科室	健康管理中心　　主要研究者：季晓东
主要研究内容	同理关爱行为是提升护理质量的基础。本研究应用情绪调节和公共服务动机理论，探讨了情绪聚焦型人力资源管理对护理人员的同理关爱行为的影响，揭示影响同理关爱行为的形成机制。调研样本量为 290 名一线护理人员，样本主要来自苏大附一院，调研主要采取线上的方式匿名进行，研究期限是 2022 年 5 月到 2022 年 12 月，研究指标主要是以情绪为中心的人力资源管理、同理关爱行为、公共服务动机和情绪劳动。研究结论不仅可以揭示情绪聚焦型人力资源管理对同情关怀行为的影响机制和情景，而且可以从理论上深化了对情绪调节能力提高过程的理解，为护理人员管理提供启示。
科研管理部门立项审查意见	同意立项。<br>签名：　　2024年09月20日
审查意见	经伦理委员会审核，该研究项目为回顾性研究，依据我国相关法律、法规和国际伦理准则，该研究方案设计科学，符合伦理原则，同意开展本项研究。
伦理委员会审核委员签字	委员：　　苏州大学附属第一医院医学伦理委员会（盖章）<br>2024 年 09 月 23 日

备注：审查文件清单见附件：附件 1：科研诚信承诺书

附件 2：研究方案

附件 3：豁免知情同意书申请
